# Treatment of reducible unstable fractures of the distal radius: randomized clinical study comparing the locked volar plate and external fixator methods: study protocol

**DOI:** 10.1186/1471-2474-15-65

**Published:** 2014-03-05

**Authors:** Jorge Raduan Neto, Vinicius Ynoe de Moraes, João B Gomes dos Santos, Flávio Faloppa, João Carlos Belloti

**Affiliations:** 1 Hand, Arm and Shoulder Surgery Unit, Department of Orthopedics and Traumatology, Federal University of São Paulo, UNIFESP/EPM, São Paulo, SP, Brazil; 2 Hand, Arm and Shoulder Surgery Unit, Rua Borges Lagoa, 778 Vila Clementino, São Paulo, SP, Brazil

**Keywords:** Distal radius fracture, Volar plate, External fixator, Randomized, Prospective (annex 1)

## Abstract

**Background:**

Various treatments are available for reducible unstable fractures of the distal radius, such as closed reduction combined with fixation by external fixator (EF), and rigid internal fixation using a locked volar plate (VP). Although there are studies comparing these methods, there is no conclusive evidence indicating which treatment is best. The hypothesis of this study is that surgical treatment with a VP is more effective than EF from the standpoint of functional outcome (patient-reported).

**Methods/Design:**

The study is randomized clinical trial with parallel groups and a blinded evaluator and involves the surgical interventions EF and VP. Patients will be randomly assigned (assignment ratio 1:1) using sealed opaque envelopes. This trial will include consecutive adult patients with an acute (up to 15 days) displaced, unstable fracture of the distal end of the radius of type A2, A3, C1, C2 or C3 by the Arbeitsgemeinschaft für Osteosynthesefragen–Association for the Study of Internal Fixation classification and type II or type III by the IDEAL^32^ classification, without previous surgical treatments of the wrist. The surgical intervention assigned will be performed by three surgical specialists familiar with the techniques described. Evaluations will be performed at 2, and 8 weeks, 3, 6 and 12 months, with the primary outcomes being measured by the Disabilities of the Arm, Shoulder and Hand (DASH) questionnaire and measurement of pain (Visual Analog Pain Scale and digital algometer). Secondary outcomes will include radiographic parameters, objective functional evaluation (goniometry and dynamometry), and the rate of complications and method failure according to the intention-to-treat principle. Final postoperative evaluations (6 and 12 months) will be performed by independent blinded evaluators. For the Student’s *t*-test, a difference of 10 points in the DASH score, with a 95% confidence interval, a statistical power of 80%, and 20% sampling error results in 36 patients per group.

**Discussion:**

Results from this study protocol will improve the current evidence regarding to the surgical treatment these fractures.

**Trial registration:**

ISCRTN09599740

## Background

Even though distal radius fractures are among the most frequent of the upper limb [1], the best treatment for these fractures remains unclear [2,3]. A wide variety of treatments have been described, including conservative treatment with immobilization by casting [4], closed reduction and fixation with percutaneous Kirschner wires (PKW) [5-7], and other diverse methods for external [7-9] and internal [10-13] fixation.

When planning treatment, determining the stability of these fractures is extremely important because stable fractures can be treated by nonsurgical methods [14]. Nonetheless, when instability is present the fractures require surgical methods of reduction and fixation, such asthey internal fixation methods [10-13], external fixation methods [7,15], or percutaneous methods [7]. At present, there is no conclusive evidence that there are any differences in the effectiveness among these methods.

Recently, the employment of locked volar plates has gained wide usage as a form of treatment allowing direct reduction of the fracture, greater stability and shorter rehabilitation time [11-13]. However, there are known disadvantages related to open fracture reduction, such as tenosynovitis, tendon rupture, and subsequent surgeries to remove the implant [9,16,17].

In contrast, methods of external fixation [7,15,17] have the advantage of being less invasive to the fracture site because the principle of indirect reduction is employed, thereby making it a more biological alternative. However, it has the disadvantage of being a method requiring a longer immobilization period, complications related to pin-track infection, failure to maintain reduction, neuritis, and longer time to recover functionality.

A study [18] comparing treatment of distal radius fractures by locked volar plate versus external fixator have shown that patients undergoing VP treatment had a better range of motion after the final treatment when compared with patients undergoing treatment with an EF. However, no functional difference was detected between the two groups of patients.

Margaliot and collaborators [17], published a systematic review of treatments for distal radius fractures, in which 28 studies with 917 patients were analyzed. The authors concluded that the advantages of osteosynthesis by locked VP are not supported by the literature when compared with an external fixator, and that randomized clinical trials, as guidance for treating such patients, are lacking. A Cochrane Collaboration systematic review [19] of the literature on surgical procedures for the treatment of distal radius fractures analyzed 48 randomized studies comparing different methods of surgical treatment, and it was concluded that insufficient scientific information was available to determine which surgical treatment method was best for this type of fracture. Regarding complications, some studies report a considerable incidence of complications with the use of VP [16], while other studies indicate greater complications with the EF method [9].

David and collaborators [20] published a systematic review of treatment for distal radius fractures that analyzed 12 studies with 1,011 patients. The authors concluded that there are an insufficient number of randomized clinical trials comparing osteosynthesis using a locked VP versus external fixation for the treatment of distal radial fractures.

Therefore, we conceived this study based on the hypothesis that the use of locked volar plates in young patients with unstable distal radius fractures will provide better results in terms of patient-reported functional outcomes. It is also anticipated that there will be a shorter time for returning to work, better radiographic parameters, and a lower rate of complications when compared to the external fixation method at the end of a one-year follow-up period. The objectives of the study are to determine which is the most effective method for treatment of young patients with unstable fractures of the distal radius: rigid internal fixation with a locked volar plate versus an external fixator combined with PKW. The primary outcomes that will be evaluated is patient-reported function via the “Disabilities of the Arm, Shoulder and Hand” (DASH) questionnaire [21] and pain [“Visual Analog Pain Scale” (VAPS) [22] and digital algometer]. The secondary outcomes that will be evaluated are as follows: radiographic parameters, objective functional evaluation (goniometry and dynamometry), and rates of complications and failures (intention-to-treat principle).

## Methods/Design

**Figure 1:**
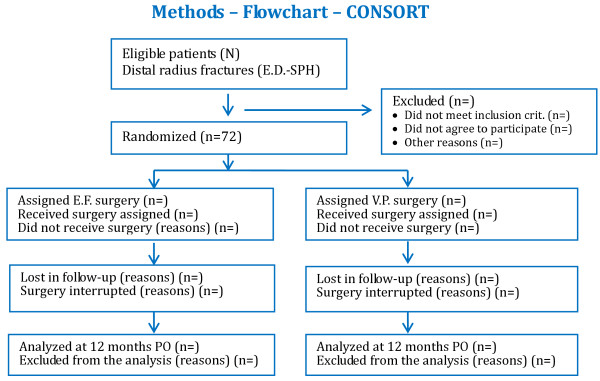
Flowchart of patients included in the study.

This research project is filed under the title “External fixation or volar plating for treating deviated distal radius fracture: randomized clinical trial” under the number, ISRCTN09599740 (http://www.controlled-trials.com/ISRCTN09599740/radius). This study was approved by the Research Ethics Committee of this institution under the number CEP-0011/11 (REC-0011/11– annex 2). The study flowchart is given in Figure 1.

1. Type and location of the study

The study is a randomized clinical trial with a blinded evaluator and is performed in the Department of Orthopedics and Traumatology – Hand, Arm, and Shoulder Surgery Unit– EPM – UNIFESP.

2. Participants

Study participants include adult patients of both sexes with acute fractures (up to 15 days) of the distal end of the radius that have not undergone prior surgical treatments and which meet inclusion criteria.

3. Inclusion criteria

### Classification

Two classifications will be utilized, the Arbeitsgemeinschaft für Osteosynthesefragen–Association for the Study of Internal Fixation (AO-ASIF) classification [23,24] and the “IDEAL” classification [25], which consists of the following (Table 1):

Type I Fracture – 0–1 point

Type II Fracture – 2–3 points

Type III Fracture – 4–5 points

**Table 1:** IDEAL classification system: rationale and scoring

	**Parameter**	**0 points**	**1 points**
I	Joint incongruity	No	Step or gap > 2 mm
D	Displacement	No	Requires reduction
E	Energy^*^	Low	High
A	Age	<60 years old	≥60 years old
L	Associated lesions^**^	Absent	Present

*Low = fall from standing height, or High = other.

**Open fracture/carpal fractures and/or instability/distal ulnar fractures.

### Reducibility criteria

Fractures will be considered displaced if they show, before manipulation, loss of at least one of the criteria below. Fractures will be considered reducible if the radiographic parameters below [16,26] are achieved after manipulation under anesthesia (he contralateral side will be used as a reference):

– Radial length – accepted loss of up to 3 mm

– Radial inclination – accepted loss of up to 8°

– Volar tilt – accepted loss of up to 15°

– Ulnar variance – accepted difference of up to 2 mm

– Articular fragment with displacement – accepted up to 2 mm

Patients in the study will include those presenting a distal radius fracture of the 23A2, 23A3, 23C1, 23C2 or 23C3 types by the AO-ASIF classification and types II and III by the IDEAL classification that are displaced in the initial x-ray and can undergo closed reduction after manipulation under anesthesia.

### Exclusion criteria

Patients presenting one or more of the following criteria will be excluded from the study:

– Patients with marginal fractures or fractures from a shearing mechanism

– Patients with irreducible fractures

– Patients with prior history of a degenerative or traumatic disorder of the affected or the contralateral wrist joint: identified from the clinical history or diagnosed by x-rays (posteroanterior or lateral view of the wrist).

– Patients with bilateral fracture, fractures exposed to or associated with tendon or neurovascular lesions

– Patient with systemic diseases or traumatic lesions associated with the facture that restrict the application of methods or the evaluation of results

– Patient with a cognitive deficit that does not allow the patient to understand the elements of the functional evaluation

– Consent Form Refusal

### Surgical intervention

#### Initial treatment

Patients will receive treatment within the institution’s distal radius fracture clinic. They will undergo a clinical and radiographic examination, with bilateral x-rays of the wrists in posteroanterior (PA) and lateral (L) views. After applying inclusion and exclusion criteria, eligible individuals will be informed regarding the nature and objective of the study, by reading the “Informed Consent Form” and will then be enrolled after signing the form. On a pre-scheduled date, the study participant will be anesthetized and undergo a trial, closed reduction of the facture. After the trial fracture reduction, radioscopy will be performed to evaluate reducibility criteria. Patients that have fractures capable of closed reduction will be randomized and treated surgically by one of the two methods of the study. Patients that do not have distal radius fractures capable of closed reduction will be excluded from the study and will receive appropriate treatment.

#### Anesthesia

Patients will undergo a supraclavicular brachial plexus block by the Winnie-Collins technique [27].

#### Method of surgical intervention

Surgery will be performed on an outpatient basis. Three surgeons will participate in the study who have been previously determined and confirmed to be familiar with the two surgical techniques used in the study. The surgical materials needed to perform the techniques will be available in the operating room for each surgery. After anesthesia, the patient will undergo manipulation and closed reduction of the fracture, and adequate reduction will be confirmed by the image provided by the image intensifier. Identification of the method to be used for each patient will be determined only after ascertaining that the fracture is reducible by the closed reduction technique, at which time the sealed envelope will be opened and the treatment technique that is to be employed will be revealed. Should the fracture not be reducible the patient will be excluded from the study.

#### Surgical techniques

Closed reduction and linear external fixator.

The linear external fixator to be used will be a radiotransparent bar with two pin fastening platforms, with one platform being proximal on the radius and the other platform distal on the second metacarpal, offered by Synthes (code – 03.304.220S). Four threaded self-drilling pins will be used with the proximal platforms that are 4.0 mm in diameter and the distal platforms that are 2.5 mm in diameter. Osteosynthesis with an external fixator will be achieved by the following surgical technique: closed reduction of the fracture by the reduction technique employing traction and contraction manipulation; confirmation of reduction with the image intensifier; a longitudinal incision of 1.5 mm in the dorsal aspect of the forearm and 8 cm proximal from the wrist joint on the longitudinal axis of the radius; exposure of the dorsal cortex of the radius by blunt dissection, introduction of the soft tissue protector positioned at a right angle to the coronal plane of the forearm; introduction of two 4.0 mm self-drilling Schanz pins with a T-handle; double 0.01 cm incisions over the dorsal aspect of the diaphysis of the second metacarpal with a 1 cm spacing between them; dissection and exposure of the dorsal cortex of the metacarpal; placement of the soft tissue protector at a right angle to the coronal plane of the hand; introduction of two 2.5 mm self-drilling Schanz pins, with a technique similar to that for the proximal pins, in the diaphyseal region of the second metacarpal. Should the fracture be intra-articular, this surgical technique may be combined with percutaneous fixation with 1.5 or 2 mm K-wires. Where dorsal comminution is present, a bone graft may be performed with bone removed from the iliac.

Open reduction with volar approach and volar locked plate.

The volar fixed-angle locking plate offered by Synthes (code −442.493) will be used with unlocked 3.5 cm screws in the cortex proximally and with 2.4 and 2.7 mm locked screws distally. Osteosynthesis with a VP will be achieved by the following surgical technique: volar incision initiated 1 cm distal to the fold of the wrist extending longitudinally in the proximal direction about 8 cm, centered over the radial flexor tendon (RFT); incision of the superficial fascia, freeing the RFT; opening of deep fascia; section of the quadrate pronator muscle 1 cm from its radial insertion with exposure of the fracture; reduction of the fracture and temporary fixation with 1.5 or 2.0 mm K-wires; confirmation with image intensifier; placement of the volar plate; proximal placement of an unlocked screw; confirmation of correct plate plate position by radioscopy; distal placement of two to five locked screws and two additional unlocked screws proximally; layer and skin closure with sutures. Where the fracture is intra-articular this may be combined with percutaneous fixation with 1.5 or 2 mm K-wires. Application of occlusive dressing and palmar forearm splint will be removed on postoperative (PO) day 14.

### Clinical outcomes

The functional and radiographic evaluation and the satisfaction protocol will be performed by professionals not directly connected to the study at the intervals provided in Table 2. For outcomes at 6 and 12 months the evaluators will be blinded to the patient assignment groups. The minimum clinical follow-up will be 12 months, with the following parameters being considered to evaluate the results:

**Table 2:** Outcomes and measurement time

	**2 W**	**8 W**	**3 M**	**6 M**	**12 M**
DASH	**X**	**X**	**X**	**X**	**X**
VAPS	**X**	**X**	**X**	**X**	**X**
Algometer	**X**	**X**	**X**	**X**	**X**
Grip	**X**	**X**	**X**	**X**	**X**
Dig. Pinch	**X**	**X**	**X**	**X**	**X**
AOM	**X**	**X**	**X**	**X**	**X**
X-rays	**X**	**X**	**X**	**X**	**X**

#### Primary outcomes

Patient-reported functional outcomes

Functional status will be evaluated by means of the DASH questionnaire (validated for the Portuguese language [28]) at the following intervals: 2 weeks PO, 8 weeks PO, 12 weeks PO, 6 months PO, and12 months PO.

Pain

Will be evaluated by means of the VAPS and an algometer [29,30] (the digital algometer used will be a FPIX 50 – Wagner Pain Test Digital Algometer), which is to be applied in the location where pain is reported by the patient, at the following times relative to treatment: 2 weeks PO, 8 weeks PO, 12 weeks PO, 6 months PO, and 12 months PO.

#### Secondary outcomes

Radiographic parameters

Measurement of angular displacements [26,31,32] and associated lesions [33]. The radial length, radial inclination, volar tilt, ulnar variance, and articular fragment will be measured and considered in the evaluation of radiographs in the posteroanterior and lateral views at the following times relative to treatment: preoperative, 2 weeks PO, 8 weeks PO, 12 weeks PO, 6 months PO, and 12 months PO. Measurements will be performed independently by two researchers on different occasions.

Objective functional evaluation

Arcs of motion will be measured for the wrist and the metacarpal-phalangeal joints of the 1st to the 5th fingers (a goniometer will be employed), palm grip strength (a “Jamar Plus – Hand Dynamometer” digital model dynamometer will be used), pulp-to-pulp, three-point and lateral pinch strength (a digital pinch dynamometer, model “Jamar Digital Pinch Gauge”, will be used), at the following times relative to treatment: 2 weeks PO, 8 weeks PO, 12 weeks PO, 6 months PO, and 12 months PO.

Complications

Any clinical situation will be considered a complication if it requires treatment by a clinical procedure or surgery not provided for in the protocol. All complications will be recorded for subsequent stratification into major and minor complications.

Method failure

Any complication will be considered a method failure if it involves an interruption or change in the treatment method from the method previously randomized.

### Statistical methods

Epidemiological data will be collected (age, sex, type of fracture, time between fracture, and treatment). Standard deviations or confidence intervals, in the case of percentages, will be provided for each type of data. As a method for confirming randomization effectiveness, data will be compared when stratified by assignment group.

The assumption of normality will be verified by the Shapiro-Wilk test for the use of parametric tests. A Pearson’s chi-square test will be employed to analyze results from the two groups involving categorical variables. A Student’s (parametric) *t*-test will be used for comparing groups of numeric variables. Paired t-tests (parametric) and Wilcoxon tests (non-parametric) will be used to compare clinical progression at intervals of 2-, 8-, and 12-weeks PO and subsequently at 6- and 12-months PO. The significance level used in all statistical tests is to be 5% (alpha = 0.05), with tests having a P value less than 0.05 being statistically significant.

Should differences be found in primary outcomes, then statistical methods will be used to test whether there is robust correlation between epidemiological factors or fracture seriousness and the observed functional outcomes. In addition, we intend to employ Kaplan-Meier survival analysis to evaluate drop-outs should high rates of complications (greater than 20%) occur in either assignment group.

Patients who experience treatment failures and require additional surgery will be monitored and their results computed in the primary assignment group (intention-to-treat principle). Provisions are to be made for blinded statistical analysis of data by a statistician who is unfamiliar with the objectives and outcomes of interest.

### Randomization and Masking

The decision to include patients in assignment groups will be made by the following randomization method: envelopes will be numbered on the outside with consecutive numbers, the assignment of the method in each envelope will be made randomly and consecutively using randomization software (http://www.randomizer.org/), and the envelope will be opened only in the operating room after verification of the fracture reducibility criterion. The randomization procedure will be delegated to a person who is not directly connected to the study.

### Sample size calculation

The sample size was calculated based on a 10 point difference (10%) in the DASH between the two groups studied, assuming a 95% confidence interval, 80% statistical power and 20% sampling error, resulting in 36 patients in each group.

## Discussion

The results from this randomized clinical study are expected to be published in December of 2015. The objective of the study is to clarify the apparent lack of conclusive evidence regarding treatment of unstable fractures of the distal end of the radius.

## Abbreviations

AO: Arbeitsgemeinschaft für osteosynthesefragen foundation; ASIF: Association for the study of internal fixation; REC: Research ethics committee; DASH: Disabilities of arm, shoulder and hand; EPM: Paulista school of medicine; VAPS: Visual analog pain scale; E.F.: External fixator; L: Lateral; PA: Posteroanterior; PO: Postoperatively; VP: Volar plate; UNIFESP: Federal University of São Paulo.

## Competing interests

The authors declare that there are no conflicts of interest.

## Authors’ contributions

JRN, VYM, and JCB developed the study protocol, obtained resources for carrying out the study, and are responsible for recruitment, performing surgical treatment, and evaluation of the patients included in this study. JBGD and FF developed the study protocol. All authors read and approved the final manuscript.

## Pre-publication history

The pre-publication history for this paper can be accessed here:

http://www.biomedcentral.com/1471-2474/15/65/prepub

## Study Financing

This study is financed by funds provided by the São Paulo State Research Support Foundation / Fundação de Amparo à Pesquisa do Estado de São Paulo (FAPESP – 2012/00806-2- annex 3).
